# Case Report: Rare gastric and bone metastases after early endocrine therapy discontinuation in hormone receptor-positive breast cancer: lessons and therapeutic insights

**DOI:** 10.3389/fonc.2026.1773152

**Published:** 2026-04-14

**Authors:** Minjun Lu, Guinv Hu, Qinyan Shen

**Affiliations:** Department of Thyroid and Breast Surgery, Dongyang People’s Hospital Affiliated to Wenzhou Medical University, Dongyang, Zhejiang, China

**Keywords:** bone metastasis, breast cancer, endocrine therapy compliance, gastric metastasis, hormone receptor-positive

## Abstract

Breast cancer with gastric metastasis is extremely rare. Non-persistence with adjuvant endocrine therapy is associated with increased recurrence/metastasis risk in hormone receptor-positive (HR+) breast cancer. A 33-year-old female was initially diagnosed with left breast cancer in July 2011, undergoing modified radical mastectomy, adjuvant chemotherapy, radiotherapy, and endocrine therapy (discontinued voluntarily after 1 year). In February 2025, she presented with low back pain and epigastric discomfort; examinations confirmed gastric and multiple bone metastases (ER+,PR+,HER2-). She received first-line endocrine therapy (letrozole+ribociclib+goserelin) combined with denosumab. Re-examination in September 2025 showed normal CEA, significantly decreased CA153, and no disease progression per RECIST 1.1. This case suggests that early endocrine therapy discontinuation may potentially contribute to late recurrence/metastasis. Breast cancer gastric metastasis diagnosis relies on medical history, gastroscopy, and immunohistochemistry (GATA3+, CK7+, CK20-). The combination regimen yielded favorable efficacy, providing clinical reference for similar rare cases.

## 1 Introduction

Breast cancer is the most common malignant tumor in women globally (1). HR+ breast cancer accounts for ~70% of cases; adjuvant endocrine therapy (ET) is pivotal to reduce recurrence (2–4). Standard adjuvant ET is 5–10 years; however, early discontinuation is common and associated with higher recurrence risk.

Breast cancer metastases most frequently involve bone, lung, liver, and brain. Gastric metastasis is extremely rare (<1%) (5, 6) and easily misdiagnosed due to non-specific symptoms (7). Here, we report a case of late gastric and bone metastases preceded by early ET discontinuation 13 years after primary diagnosis. This case does not establish causality but illustrates a clinical association and provides insights into diagnosis and treatment of rare metastatic sites.

## 2 Case presentation

### 2.1 Patient and primary tumor summary (2011)

A 33-year-old premenopausal female, no comorbidities, no family history of cancer, non-smoker, non-drinker.

### 2.2 Primary treatment (2011–2012)

Surgery: Left breast modified radical mastectomy + latissimus dorsi myocutaneous flap reconstruction. Primary tumor characteristics are detailed in **Table 1**.

**Table 1:** Primary tumor characteristics (2011).

Item	Details
Histology	High-grade DCIS with focal invasion
Invasive focus size	High-grade DCIS with focal invasion
Invasive focus size	≤1 mm (microinvasion)
Number of invasive foci	1 focus
Axillary nodes	12 examined; 0 positive
pT stage	pT1mi
pN stage	pN0
Surgical margins	Negative
ER (2011)	Weakly positive, Allred score 3–4
PR (2011)	Negative
HER2 (2011)	IHC 2+; FISH not performed in 2011
Ki-67	<10%
E-Cadherin	Strongly positive

#### 2.2.1 Chemotherapy

6 cycles of cyclophosphamide (CTX) 700 mg d1 + fluorouracil (5-FU) 0.7 g d1 + epirubicin 70 mg d1–2.

#### 2.2.2 Rationale

In 2011, young age (33 years) and focal invasion were considered relative indications for adjuvant chemotherapy per Chinese and ASCO guidelines at that time, despite microinvasion and node-negative status.

#### 2.2.3 Radiotherapy

Left chest wall + supra/infraclavicular region DT 50Gy/25f; tumor bed boost DT 10Gy/5f.

#### 2.2.4 Endocrine therapy

Tamoxifen 10 mg bid + goserelin 3.6 mg monthly. The patient voluntarily discontinued all ET after 1 year.

#### 2.2.5 Patient perspective on discontinuation

The patient reported no severe treatment-related toxicity during ET. She discontinued mainly due to concerns about future fertility and psychosocial stress, with insufficient understanding of long-term recurrence risk despite repeated physician counseling.

### 2.3 Recurrent presentation (February 2025)

2-week persistent low back pain + epigastric discomfort.

#### 2.3.1 Breast/chest wall evaluation

Ultrasound and chest CT showed no local recurrence or new primary breast cancer.

### 2.4 Imaging protocol and key findings

Chest/abdominal CT: Contrast−enhanced; no visceral metastases except for gastric mucosal abnormality.

#### 2.4.1 Whole-body bone scan

**Figure 1:**
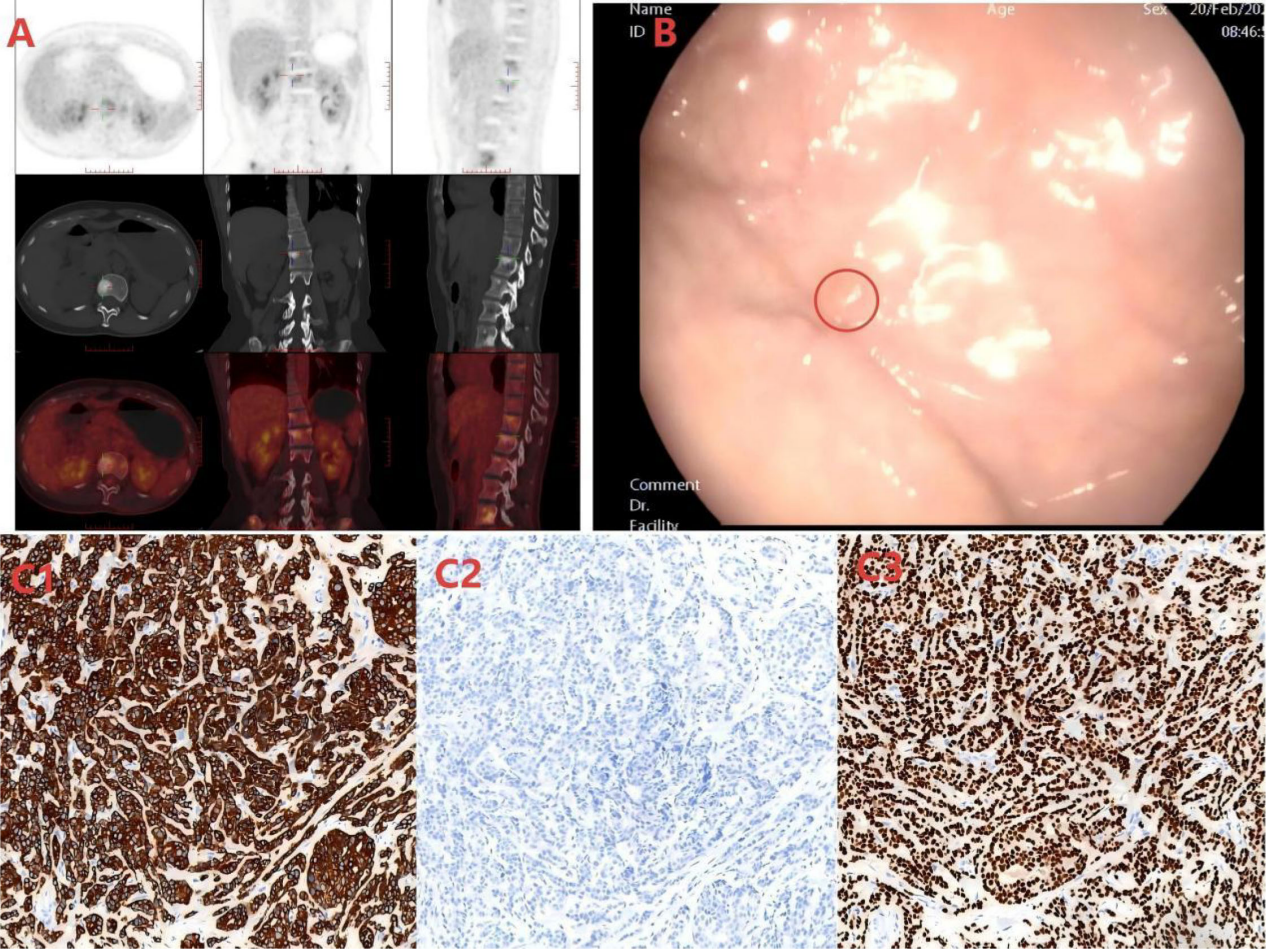
**(A)** Whole-body bone scan shows abnormally increased radioactive uptake in multiple systemic bones, suggesting multiple systemic bone metastases. **(B)** A polypoid elevation with a diameter of approximately 0.4 cm in the gastric fundus is shown in the red circle under gastroscopy. **(C1)** Immunohistochemical results of the biopsy of the gastric fundus polyp indicate positive staining for CK7.(20×). **(C2)** Immunohistochemical findings of the gastric fundus polyp biopsy indicate negative staining for CK20.(20×) **(C3)** Immunohistochemical findings of the gastric fundus polyp biopsy indicate positive staining for GATA-3.(20×).

99mTc-MDP planar imaging; widespread increased uptake in ribs, scapulae, sternum, spine, pelvis, femurs. (**Figure 1A**).

#### 2.4.2 PET/CT

18F−FDG; multiple bone metastases with moderate uptake; no other distant metastases; SUVmax of bone lesions ranged 3.5–6.2.

### 2.5 Gastroscopy and pathology

Gastroscopy: 0.4 cm polypoid lesion at gastric fundus (**Figure 1B**). Biopsy showed atypical cell nests consistent with breast cancer metastasis.

#### 2.5.1 IHC of gastric metastasis

CK(AE1/AE3)(+), CgA(-), Syn(-), CD56(-), GATA3(+), ER (strongly positive, ~90%), PR (strongly positive, ~20%), HER2 IHC 2+; FISH negative, CAM5.2(+), CK20(-), CK7(+), Ki-67 (~15%).(**Figures 1C1-C3**).

#### 2.5.2 Bone metastasis

No biopsy performed; diagnosis justified by typical distribution, intense uptake on bone scan/PET-CT, and clinical response to anti-bone metastasis therapy.

### 2.6 Diagnosis

HR+/HER2- recurrent/metastatic breast cancer with gastric metastasis + multiple bone metastases (AJCC 8th stage IV).

### 2.7 Treatment regimen

Letrozole 2.5 mg qd po.

Goserelin 3.6 mg s.c. q4w.

Ribociclib 400 mg qd (200 mg bid) po, 3 weeks on/1 week off.

Denosumab 120 mg s.c. q4w.

#### 2.7.1 Adverse events

No grade ≥3 AEs; mild fatigue and nausea observed.

### 2.8 Follow-up and outcomes

Treatment start: 2025−03−10.

Reassessment: 2025−09−12 (6.1 months).

CEA: 15.6 → 3.2 ng/mL (normal).

CA153: 89.3 → 35.8 U/mL.

#### 2.8.1 Assessment

No progressive disease (PD) per RECIST 1.1; symptoms markedly relieved. The patient's diagnosis and treatment timeline is detailed in **Table 2**.

**Table 2:** Timeline table (2011–2025).

Time	Event
2011-07	Primary breast cancer diagnosis & surgery
2011-08 — 2012-01	Adjuvant chemotherapy
2012-02 — 2012-03	Radiotherapy
2012-04 — 2013-03	Endocrine therapy (tamoxifen+ goserelin)
2013-04	ET discontinued
2013— 2024	Regular follow-up; no recurrence
2025-02	Symptoms (back pain + epigastric discomfort)
2025-02— 2025-03	Diagnosis of gastric + bone metastases
2025-03	Start target regimen
2025-09	Reassessment: non-progressive disease

## 3 Discussion

### 3.1 Endocrine therapy non−persistence and late recurrence

Adjuvant ET is the cornerstone for HR+ breast cancer. Early discontinuation is associated with higher recurrence risk (8–10). In this patient, 1−year ET preceded late distant metastasis at 13 years. Causality cannot be proven; tumor dormancy, residual micro−disease, and baseline biological risk may also contribute.

### 3.2 Diagnosis of gastric metastasis

Gastric metastasis from breast cancer is rare (<1%). Diagnosis depends on history, endoscopy, and IHC: GATA3+, CK7+, CK20– is highly suggestive of breast origin (11–13). Primary gastric cancer more frequently shows a CK7−/CK20+ profile, though exceptions exist. For breast cancer patients with gastrointestinal symptoms, early gastroscopy and IHC are critical to avoid misdiagnosis.

### 3.3 Therapeutic efficacy

For premenopausal HR+/HER2- advanced breast cancer, AI + CDK4/6 inhibitor + OFS is a standard first-line option (14, 15). Denosumab effectively prevents skeletal-related events (16). This patient achieved durable disease control with favorable tolerance.

## 4 Limitations

1. This is a single case; causality between early ET discontinuation and metastasis cannot be established.
2. Some primary tumor details (e.g., 2011 HER2 FISH) are unavailable.
3. Bone metastases were diagnosed by imaging rather than histopathology.
4. Long-term survival outcomes remain to be observed.

## 5 Conclusions

Early discontinuation of adjuvant endocrine therapy is associated with increased risk of late recurrence/metastasis in HR+ breast cancer. Breast cancer gastric metastasis is rare and relies on history, gastroscopy, and IHC (GATA3+, CK7+, CK20–). For HR+/HER2- metastatic breast cancer with gastric and bone involvement, letrozole + ribociclib + goserelin plus denosumab achieves favorable short-term efficacy. This case highlights the importance of adherence support and standardized diagnosis of rare metastases.

## Data Availability

The original contributions presented in the study are included in the article/supplementary material. Further inquiries can be directed to the corresponding author.
